# *Cladophialophora bantiana* Cerebral Phaeohyphomycosis Complicated by Pulmonary Nocardiosis: A Tale of Two Infections

**DOI:** 10.1155/2019/4352040

**Published:** 2019-04-17

**Authors:** Muhammad Farhan Khaliq, Rayan E. Ihle, Christopher P. Schirtzinger

**Affiliations:** ^1^Department of Internal Medicine, Charleston Area Medical Center, Charleston, WV, USA; ^2^Department of Pulmonary Critical Care, West Virginia University, Charleston Division, Charleston, WV, USA; ^3^Department of Infectious Disease, Charleston Area Medical Center, Charleston, WV, USA

## Abstract

*Cladophialophora bantiana*, a melanized neurotropic fungus, is the most commonly reported agent of cerebral phaeohyphomycosis. We present a case of cerebral phaeohyphomycosis due to *C. bantiana* with a concomitant *Nocardia* infection in the lung. The patient was a 64-year-old male who presented with one-week history of productive cough, confusion, and staggering gait. Brain MRI showed multiple enhancing masses, and chest CT demonstrated multifocal consolidation. To confirm diagnosis, brain biopsy was performed that showed *Cladophialophora bantiana*. Bronchoscopic lung biopsy confirmed infection with *Nocardia araoensis*. The patient was treated with trimethoprim-sulfamethoxazole, meropenem, voriconazole, and liposomal amphotericin in addition to partial resection of the brain mass. After several weeks in the hospital and deteriorating status with poor prognosis, medical care was withdrawn. *Cladophialophora bantiana* infection is rare and requires multidisciplinary approach for accurate diagnostic confirmation. Aggressive and long-term treatment with voriconazole along with early neurosurgical intervention may offer an improved chance of survival in these patients.

## 1. Introduction

Phaeohyphomycosis is the collective term for infections caused by dematiaceous or darkly pigmented fungi. They derive their black color by generating melanin in their cell walls. *Cladophialophora bantiana* (*C. bantiana*) is the most commonly encountered agent to cause cerebral phaeohyphomycosis and is previously known as *Cladosporium trichoides*, *Xylohypha bantiana*, and *Xylohypha emmonsii* [1, 2]. It belongs to the order Chaetothyriales (ascomycete). Other commonly encountered isolates of the class include *Rhinocladiella mackenziei* and *Exophiala dermatitidis* that have reported to cause cerebral infection. Cases of *C. bantiana* are reported worldwide though a predominance for warmer climates with high humidity is apparent. The fungus can infect both immunocompetent and immunocompromised hosts. A high index of suspicion is needed for diagnosis with brain biopsy required for confirmation of phaeohyphomycosis. Currently, lack of evidence and management guidelines makes standardized treatment challenging. However, aggressive management with surgical resection and long-term antifungal agents are associated with improved outcomes in some reports [3]. Despite this approach, mortality reaches up to 70% [2]. We report a seemingly immunocompetent patient who developed cerebral phaeohyphomycosis but died due to secondary complications.

## 2. Case Presentation

A 64-year-old male, chronic smoker, construction worker, and avid hunter presented with a one-week history of confusion and staggering gait leading to a fall. He also complained of cough productive with black sputum. Family and travel histories were unremarkable; however, social history was remarkable for a recent devastating flood in the town where he lived that sustained a large amount of water and mud damage. On presentation, his vital signs were normal. On examination, the patient was alert but not oriented. On chest auscultation, decreased breath sounds were noted in the left lower lobe. Neurological, cardiovascular, and abdominal examinations were otherwise within normal limits. His initial set of labs revealed mild leukocytosis, a negative procalcitonin, and hyponatremia. Chest X-ray (CXR) demonstrated left lower lobe pneumonia. With this information, he was therefore thought to have community-acquired pneumonia and was initiated on azithromycin 500 mg intravenous (IV) daily, ceftriaxone 1 g IV daily, and IV hydration.

**Figure 1:**
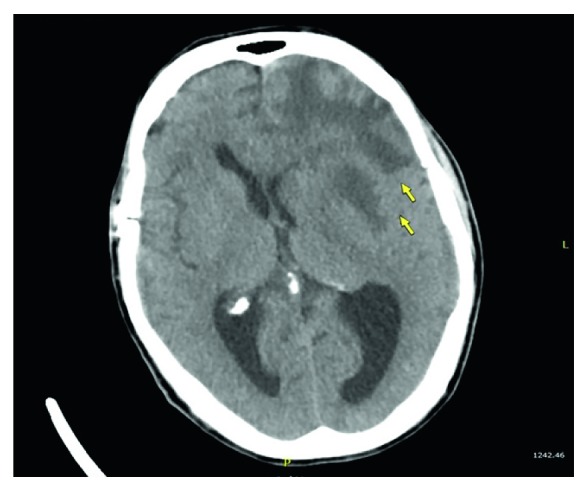
CT brain axial view demonstrating left frontal lobe mass concerning for glioblastoma.

**Figure 2:**
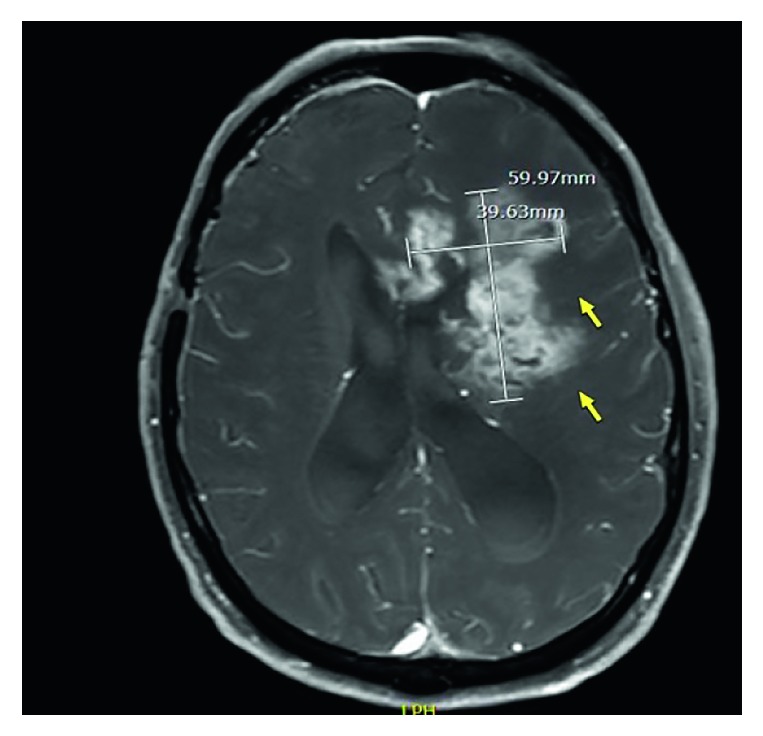
MRI brain axial view demonstrating multiple enhancing masses coalescing into a larger mass with surrounding edema resulting in midline shift involving anterior corpus callosum.

**Figure 3:**
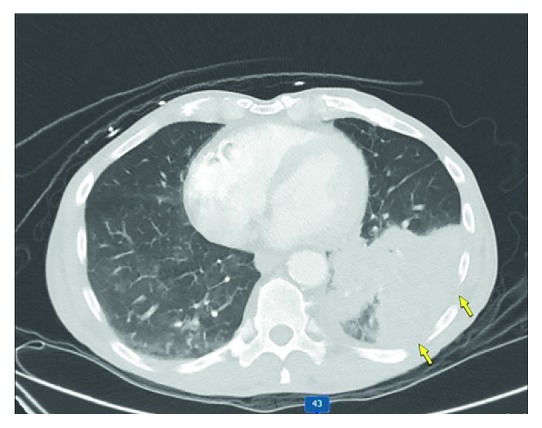
CT chest demonstrating multifocal pneumonia in the left lung base with areas of suspected internal necrosis. No mediastinal lymphadenopathy is visible.

Due to his initial neurologic complaints, a brain computed tomography (CT) was completed (Figure 1), demonstrating a left frontal mass with surrounding cerebral edema and midline shift. Brain magnetic resonance imaging (MRI) (Figure 2) described the lesion as a conglomeration of multiple left frontal lobe enhancing masses causing midline shift. This prompted the differentiation of metastatic malignancy versus glioblastoma. Dexamethasone 4 mg IV every 6 hours and levetiracetam 1500 mg IV twice daily were started after neurosurgical evaluation. CT of the chest (Figure 3) was performed for further characterization of the lung finding on CXR with the possibility of malignancy in question. It demonstrated a multifocal mass-like area of consolidation in the left lower lobe with necrosis and cavitation interpreted as a low suspicion for malignancy with close interval follow-up recommended (Figure 3). Pulmonology was consulted for evaluation with the consideration of bronchoscopy.

**Figure 4:**
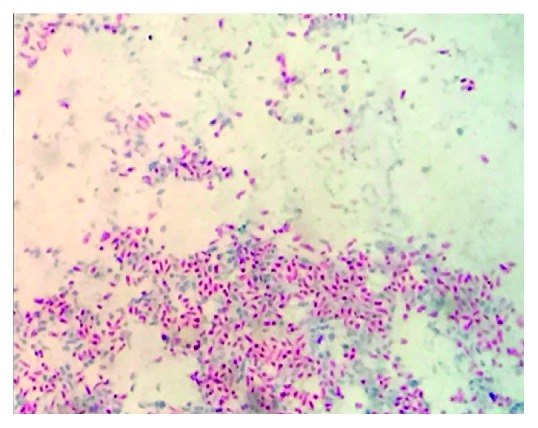
*Nocardia araoensis* growing on cultures obtained from lung biopsy at 100x magnification stained with Kinyoun acid-fast stain.

With the possibility of the brain lesion still being a glioblastoma, a stereotactic brain biopsy was performed first instead of a bronchoscopy. This biopsy revealed numerous light brown, long sparsely branching septated hyphae with dilated spores positive on Gomori methenamine silver (GMS) stain and periodic acid-Schiff (PAS) light green stains. Cultures were unfortunately not sent from this tissue at this time. Based on these pathology results, infectious disease consultation was sought, and empiric liposomal amphotericin 5 mg/kg IV daily with concurrent voriconazole 300 mg IV every 12 hours was initiated. At this time with a possible disseminated fungal disease on the differential, bronchoscopy was pursued. There were no endobronchial lesions present. Fungal culture of lung biopsies was completed at 35°C and revealed *Nocardia*. It was further speciated with matrix-assisted laser desorption-ionization time-of-flight mass spectrometry (MALDI-TOF MS) and identified as *Nocardia araoensis* (Figure 4). The patient was therefore treated with trimethoprim-sulfamethoxazole 1600 mg/280 mg orally every 6 hours and meropenem 2 grams IV every 8 hours. Blood cultures remained negative throughout his hospital course.

**Figure 5:**
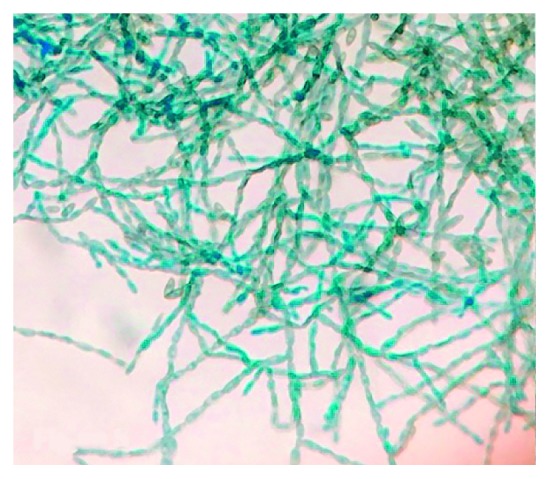
*Cladophialophora bantiana* showing septate hyphae with branching at 40x magnification from brain biopsy.

**Figure 6:**
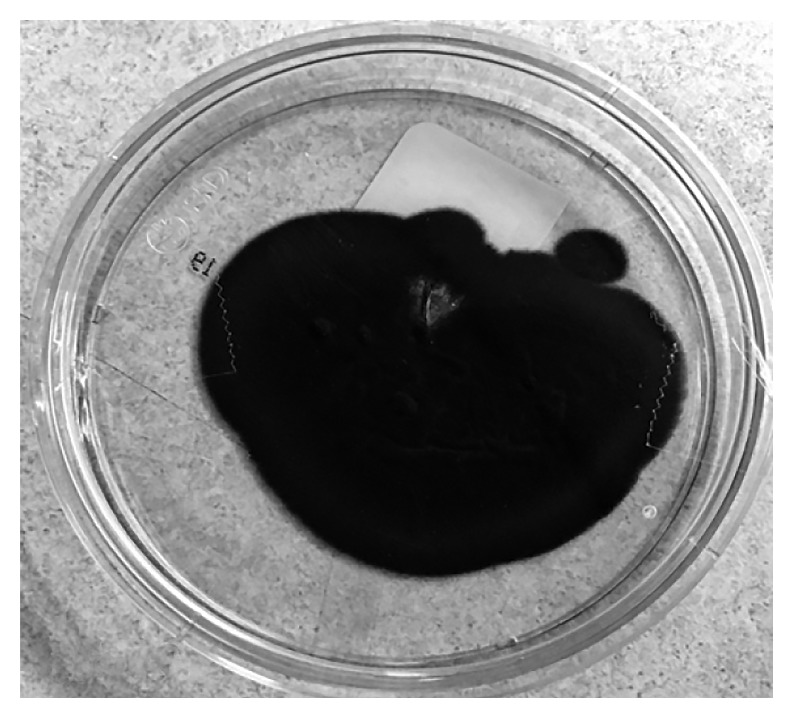
*Cladophialophora bantiana* growing on Sabouraud Agar from brain biopsy demonstrating velvety-textured olive-colored colonies.

With the still unidentified fungal brain abscess and the size of the lesion portending a poor prognosis if treated only medically, neurosurgery team performed a craniotomy with the intention of complete resection. Unfortunately, only a partial resection was achievable due to the location and size of the mass. Fungal culture of the brain tissue was completed on BBL™ Sabouraud Dextrose Agar, BBL™ Inhibitory Mold Agar, and BBL™ Brain Heart Infusion Agar with 5% Sheep Blood (BD Biosciences: Becton, Dickinson and Company, Sparks, MD) with mold isolated after 6 days after inoculation at 35°C. The isolates were described as velvety green-brown in color with very long chain length, minimal branching, and oval conida. Visual identification was made as *Cladophialophora bantiana* (Figures 5 and 6) after 20 days, referencing Larone's “Medically Important Fungi” text [1]. The patient was treated with concurrent liposomal amphotericin B and IV voriconazole for the first 5 days and then voriconazole alone due to its better bioavailability and central nervous system (CNS) penetration with lesser side effects.

The presence of two rare infections in an otherwise immunocompetent individual prompted an immunological evaluation including immunological levels of Immunoglobulin (Ig) A, E, G, and M, Herpes Simplex virus PCR, CD4 counts, T4/T8 ratio, B cell count, and Natural killer cell count and however did not reveal a cause of low IgG. He was therefore treated with human IV immunoglobulin (IVIG) Octagam® 5% 500 mg/kg IV. His 6 week hospital course was complicated by cachexia, physical deconditioning, poor neurological status, and aspiration pneumonia with recurrent episodes of sepsis. Considering his poor overall state of health and grim prognosis with incomplete resection, palliative services were consulted and family decided to withdraw further medical care and transfer to hospice for comfort care.

## 3. Discussion

Fungal infections account for less than 2% of brain abscesses [4]. Cerebral phaeohyphomycosis refers to infection of the brain caused by “dematiaceous” or melanin-producing fungi. Melanin deposited in their cell wall serves as a virulence factor and aids in evasion from the host's immunological response [5, 6]. These organisms are neurotropic and have a propensity to cause cerebral abscesses. *Cladophialophora bantiana* is the most common isolate reported globally [7]. It belongs to the order Chaetothyriales (ascomycete). Other commonly encountered isolates of the class include *Rhinocladiella mackenziei* and *Exophiala dermatitidis* that have reported to cause cerebral infection. Cases of *C. bantiana* are reported worldwide though a predominance for warmer climates with high humidity is apparent. Interestingly, this mold affects slightly more immunocompetent than immunocompromised hosts [3, 8, 9]. Males are more often affected with a mean age of 40 years [6, 8].

Several environmental factors and nature of occupation appear to have some role, and the pathogen commonly inoculates through the skin after laceration. Inhalation of spores, direct extension from paranasal sinuses, and penetrating trauma to the brain are among the other suggested modes of infection [9]. There has been no ethnic predilection reported; however, areas of warm humid climate have higher incidence [10]. Indeed, our patient's frequent outdoor exposure and the town's recent flood damage with exposure to humid, muddy conditions with the decaying plant material were likely his risks of exposure as there were no wounds found on our patient. He was also infected with Nocardia, a ubiquitous pathogen found mostly in soil, which strengthens the role of environmental exposures in this case. Our patient had no signs of soft tissue injury or infection, and respiratory cultures obtained via bronchoscopy were negative for the organism as were blood cultures. Hematogenous spread is a reasonable assumption in this gentleman after inhalation of the organism, given the presence of multiple masses differing from the presentation expected in traumatic or direct extension in which a solitary brain abscess would be found.

In fact, the most common manifestation of CNS phaeohyphomycosis is a solitary brain abscess which occurs in approximately two-thirds of cases, but multiple easily identifiable abscesses can also be seen on CT scan or MRI [8]. The lesions demonstrated on our patient's MRI are consistent with findings reported in other studies [3, 11]. Common symptomatic manifestations include headache, seizures, behavior changes, fever, and neurological and behavioral deficits. Moreover, patients may present with insidious headaches and slowly evolving neurological signs. These reported findings are consistent with our patient's presentation of slowly evolving confusion, staggering gait, and ultimately altered consciousness. In a review of the literature, the mean duration of diagnosis after development of symptoms were reported to be 115 days [8]. Indeed, in this case, definitive diagnosis of *Cladophialophora* was made after 41 days with fungal abscess determined well before.

Radiographically, *C. bantiana* abscess cannot be differentiated with certitude from bacterial abscesses, metastatic tumors, or primary tumors such as high-grade gliomas and can result in a delay of diagnosis [12]. This case presented in West Virginia has the second highest prevalence of lung cancer nationwide [13]. Often, these lung cancer cases present in later stages with metastasis to the brain. The resemblance of this patient's dual lung and brain lesions to lung cancer with cerebral metastasis, along with the patient's risk factor of prolonged tobacco use, contributed to a delay in diagnosis. Because of this diagnostic bias, initial biopsy samples were only sent for pathology. This missed opportunity of collecting culture data also further delayed definitive diagnosis until more tissue was obtained.

Despite the opportunistic nature of fungal infections, more than half of patients affected with primary cerebral phaeohyphomycosis are immunocompetent individuals [3, 8, 9]. These patients often only have involvement of the brain as in our patient, whereas patients with underlying risk factors, such as transplantation, corticosteroid therapy, diabetes mellitus, or neutropenia, have disseminated infection with widespread involvement. The presence of two rare infections in our case does suggest that a weakened cell-mediated immune response and subsequent immunocompromised state were a mediating factor for our patient. Upon evaluation, he was later found to have low immunoglobulin levels confirming this risk. It is particularly difficult to establish the diagnosis of cerebral phaeohyphomycosis in an immunocompromised host where opportunistic infections and malignancies like lymphoma become more likely etiologies. Cerebral biopsy with histological studies and exhaustive microbiological cultures for bacteria, mycobacteria, and fungi are considered the diagnostic standard and should always be performed even when an infectious etiology is not high on the differential, as illustrated above.

*Cladophialophora* species are prone to identification difficulties due to high degree of phenotypic similarities [10, 14]. *C. bantiana* does have several characteristics on culture that can lend to identification [1, 14, 15]. However, it is crucial to identify specific species as significant differences in pathogenicity, and virulence has been reported within the closely related species. Newer methods such as rolling circle amplification (RCA) that target species-specific padlock probes for the internal transcribed spacer regions of rDNA and matrix-assisted laser desorption-ionization time-of-flight mass spectrometry (MALDI-TOF MS), based on the detection of mass-to-charge (*m/z*) ratios of protonated ions from ribosomal proteins, have proven to be a timely, accurate, and reproducible method for the identification of different species [16]. In the current case, *C. bantiana* was identified based on morphologic characteristics described in Larone's Medically Important Fungi text [1]. Molecular and proteomic testing were not performed to confirm specific speciation and should be performed when possible. Nonetheless, antifungal therapy for treatment in all phaeohyphomycoses is largely the same and based on clinical experience with antifungal agents and surgical intervention.

Mortality rate of cerebral phaeohyphomycosis is 70% or more despite aggressive measures including neurosurgical intervention and long-term systemic antifungal therapy [3, 8, 9]. There has not been a significant difference in survival noted with the use of any specific antifungal agent; however, one study did show significantly improved outcomes with the combination of amphotericin, flucytosine, and itraconazole used concurrently [3, 8]. One study evaluating the *in vitro* activity of antifungals found that itraconazole, posaconazole, and isavuconazole had the lowest minimum inhibitory concentrations (MICs) and were generally more active than amphotericin [10]. Amphotericin is also less preferred due to its poor penetration into the CNS, electrolyte disturbances, and renal toxicity [17, 18]. Another study also demonstrated low MICs against all azoles except fluconazole with mixed results for amphotericin [8]. Flucytosine susceptibility was not tested in either of those studies. Echinocandins, despite a low-side effect profile and lesser drug-drug interaction, demonstrate no susceptibility against these organisms with consistently high MICs [2, 8]. The duration of the treatment varies for an individual patient, and there is no consensus about how long the course of treatment should be.

Although invasive treatment of bacterial brain abscesses has evolved in an era of minimally invasive neurosurgical management, surgical resection is still recommended as adjunctive therapy in brain abscesses particularly in fungal infections [17, 19, 20]. As antifungal penetration may be particularly low in areas of necrosis, removal of microbial burden of potentially viable fungi may aid in recovery [18]. Some studies suggest that full surgical resection may be associated with improved survival rates as compared to partial resection or aspiration alone [3, 5, 8, 21]. Lastly, it is essential to note that residue requires proper handling and adherence to care of surgical instruments as *C. bantiana* is on the Biosafety Level 2 containment list [5]. Preventing infection of new individuals is of utmost importance, given the very high mortality and difficulties in diagnosis and treatment as discussed above.

## 4. Conclusions

Several distinctive aspects exist to our case. The presence of both lung and brain masses in a high-risk patient in an area of high lung cancer incidence and with radiographic characteristics of glioblastoma with possible pneumonia prompted only consideration of malignancy on the differential of the brain abscess. This diagnosis was delayed by not sending culture data from the initial brain biopsy. The presence of a completely separate lung infection also contributed to delay. Based on the limited existing evidence and experience from our case, it is easy to conclude that outcomes depend on early and accurate diagnosis. We suggest multidisciplinary approach involving neurology, neurosurgery, infectious disease, microbiology, and pathology for accurate diagnostic confirmation and minimizing delay. Confirmatory molecular testing of molds is now recommended for accurate identification and susceptibility testing. Multimodal therapy with two to three antifungals along with surgical resection may improve outcomes. However, there is a paucity of data and guidelines to determine a standardized approach to infections from these rare black molds. Cases should be handled with care not only for proper diagnosis but also for prevention of infecting individuals involved in the patient's case.

## Abbreviations

CNS: Central nervous system
CT: Computed tomography
CXR: Chest X-ray
GMS: Gomori's methenamine silver
IV: Intravenous
IVIG: Intravenous immunoglobulin
MALDI-TOF MS: Matrix-assisted laser desorption-ionization time-of-flight mass spectrometry
MIC: Minimum inhibitory concentrations
MRI: Brain magnetic resonance
PAS: Periodic acid-Schiff
PCR: Polymerase chain reaction
RCA: Rolling circle amplification.

## References

[1] Larone D. H. (2011). *Medically Important Fungi: A Guide to Identification*.

[2] Garzoni C., Markham L., Bijlenga P., Garbino J. (2008). *Cladophialophora bantiana*: a rare cause of fungal brain abscess. Clinical aspects and new therapeutic options. *Medical Mycology*.

[3] Revankar S. G., Sutton D. A., Rinaldi M. G. (2004). Primary central nervous system phaeohyphomycosis: a review of 101 cases. *Clinical Infectious Diseases*.

[4] Brouwer M. C., van de Beek D. (2017). Epidemiology, diagnosis, and treatment of brain abscesses. *Current Opinion in Infectious Disease*.

[5] Ahmad M., Jacobs D., Wu H. (2017). *Cladophialophora bantiana*: a rare intracerebral fungal abscess-case series and review of literature. *Surgery Journal*.

[6] Gandham P. (2014). *Cladophialophora bantiana*. *International Journal of Research in Medical Sciences*.

[7] Atul A., Vichal R. (2015). A case of cerebral abscess due to *Cladophialophora bantiana*. *Journal of Microbiology and Infectious Disease*.

[8] Chakrabarti A., Kaur H., Rudramurthy S. M. (2016). Brain abscess due to *Cladophialophora bantiana*: a review of 124 cases. *Medical Mycology*.

[9] Kantarcioglu A. S., Hoog G. S. (2004). Infections of the central nervous system by melanized fungi: a review of cases presented between 1999 and 2004. *Mycoses*.

[10] Badali H., de Hoog G. S., Curfs-Breuker I., Klaassen C. H. W., Meis J. F. (2010). Use of amplified fragment length polymorphism to identify 42 cladophialophora strains related to cerebral phaeohyphomycosis with in vitro antifungal susceptibility. *Journal of Clinical Microbiology*.

[11] Harrison D. K., Moser S., Palmer C. A. (2008). Central nervous system infections in transplant recipients by *Cladophialophora bantiana*. *Southern Medical Journal*.

[12] Hakyemez B., Erdogan C., Yildirim N., Parlak M. (2005). Glioblastoma multiforme with atypical diffusion-weighted MR findings. *The British Journal of Radiology*.

[13] Siegel R. L., Miller K. D., Jemal A. (2018). Cancer statistics 2018. *CA: A Cancer Journal for Clinicians*.

[14] Ajantha G. S., Kulkarni R. D. (2011). *Cladophialophora bantiana*, the neurotropic fungus–a mini review. *Journal of Clinical Diagnosis and Research*.

[15] Matsumoto T., Ajello L., Ajello L., Hay R. F. (1998). Agents of phaeohyphomycosis. *Medical Mycology*.

[16] Cañete-Gibas C. F., Wiederhold N. P. (2018). The black yeasts: an update on species identification and diagnosis. *Current Fungal Infection Reports*.

[17] Aljuboori Z., Hruska R., Yaseen A., Arnold F., Wojda B., Nauta H. (2017). Fungal brain abscess caused by “Black Mold” (*Cladophialophora bantiana*)—a case report of successful treatment with an emphasis on how fungal brain abscess may be different from bacterial brain abscess. *Surgical Neurology International*.

[18] Schwartz S., Kontoyiannis D. P., Harrison T., Ruhnke M. (2018). Advances in the diagnosis and treatment of fungal infections of the CNS. *The Lancet Neurology*.

[19] Brouwer M. C., Tunkel A. R., McKhann G. M., van de Beek D. (2014). Brain abscess. *New England Journal of Medicine*.

[20] https://www.atsjournals.org/doi/abs/10.1164/ajrccm-conference.2018.197.1_MeetingAbstracts.A5474

[21] Roche M., Smyth E. (2005). A case of septic arthritis due to infection with Gemella morbillorum. *Journal of Infection*.

